# Mepitel Film is superior to Biafine cream in managing acute radiation‐induced skin reactions in head and neck cancer patients: a randomised intra‐patient controlled clinical trial

**DOI:** 10.1002/jmrs.397

**Published:** 2020-05-31

**Authors:** Jing Yan, Ling Yuan, Juan Wang, Shuangshuang Li, Mengdi Yao, Kongcheng Wang, Patries M. Herst

**Affiliations:** ^1^ Comprehensive Cancer Centre Drum Tower Hospital/Clinical Cancer Institute of Nanjing University Nanjing China; ^2^ Department of Radiation Therapy University of Otago Wellington New Zealand

**Keywords:** Mepitel film, radiation‐induced skin reactions, head and neck cancer patients, RISRAS, RTOG

## Abstract

**Introduction:**

We previously showed that Mepitel Film decreased the severity of acute radiation‐induced skin reactions in head and neck cancer patients. In the current study, we compared the effect of Mepitel Film and Biafine cream on skin reaction severity in a larger cohort of head and neck cancer patients.

**Methods:**

A total of 44 head and neck cancer patients were recruited with 39 patients contributing full data sets for analysis. Patients received a dose of 50 Gy in 25 fractions to the bilateral lymph nodes in the neck. Left and right lymph node areas were randomised to either Mepitel Film or Biafine cream, applied prophylactically. Skin reaction severity was measured using Radiation‐Induced Skin Reaction Assessment Scale (RISRAS) and expanded Radiation Oncology group (RTOG) grades. Skin dose was measured using gafchromic Film.

**Results:**

Skin reaction severity (combined RISRAS score) underneath Mepitel Film was decreased by 30% (*P* < 0.001) and moist desquamation rates by 41% (*P* < 0.001). Skin dose underneath Mepitel Film and Biafine cream was similar (*P* = 0.925) and unlikely to have affected skin reaction severity. The vast majority (80%) of patients preferred Mepitel Film over Biafine cream. Negative aspects of Mepitel Film included poor adherence (11/39) and discomfort (16/39) during hot weather and showering and itchy skin underneath Mepitel Film (12/39).

**Conclusions:**

Mepitel Film was superior to Biafine cream in reducing the severity of acute radiation‐induced skin reactions and moist desquamation incidence in our head and neck patient cohort.

## Graphical abstract

Mepitel Film, compared with Biafine cream, significantly decreased the severity of acute radiation‐induced skin reactions by 30% and moist desquamation rates by 41% in our cohort of 39 head and neck cancer patients.

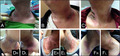

## Introduction

Patients with breast cancer or head and neck cancer can develop severe acute radiation‐induced skin reactions because their skin receives a relatively high dose as these tumours are close to the skin. With the exception of corticosteroids in breast cancer patients,^1^, ^2^, ^3^, ^4^ many different pharmacological topical interventions, dressings and systemic treatments have failed to consistently decrease skin reaction severity over and above standard care in randomised clinical trials in breast cancer patients^5^, ^6^, ^7^, ^8^, ^9^, ^10^, ^11^, ^12^ and head and neck cancer patients.^13^, ^14^

Our group has focused on protecting the radiation‐damaged skin from further friction damage using soft silicone dressings (recently reviewed by^15^). We found that, in breast cancer patients, Mepitel Film decreased acute radiation‐induced skin reactions by up to 40% when used in a management context from the moment faint erythema is visible^16^, ^17^ or by > 90% when used prophylactically from the first day of radiation therapy.^18^ A recent multicentre Danish study with Mepitel Film in breast cancer patients, using a similar intra‐patient‐controlled design, also reported significantly lower levels of pain, burning, itchiness as well as decreased sensitivity and oedema in Mepitel Film‐covered skin. RTOG scores by independent blinded observers revealed a statistically significant decrease in skin reaction severity in favour of Mepitel Film in mastectomy patients and in patients treated with a total dose of 50Gy on the last day of radiation therapy.^19^

Another patient cohort that is likely to benefit from better management of radiation‐induced skin reactions are head and neck cancer patients who face many challenges in addition to skin reactions and their quality of life during radiation therapy is generally extremely poor^13^, ^14^. We therefore conducted a feasibility study with Mepitel Film in head and neck cancer patients in New Zealand (*n* = 22) and China (*n* = 11).^20^ We showed that Mepitel Film decreased overall skin reaction severity by just under 30%. Our feasibility study also showed that Mepitel Film did not adhere well in men with strong beard stubble in the neck area, but this was not the case in Chinese men who do not have this issue. Therefore, for the current study, we added datasets from 28 Chinese patients to those of the 11 Chinese patients from the feasibility study to determine whether or not Mepitel Film is superior to standard care in managing radiation‐induced skin reactions in head and neck cancer patients. Our two main objectives were to compare the effect of Mepitel Film and Biafine cream on (1) overall skin reaction severity and (2) on the rates of moist desquamation.

## Methods

This is a randomised, intra‐patient controlled open‐label stage II clinical trial, comparing the effects of Mepitel Film against those of Biafine cream on the severity of acute radiation‐induced skin reactions in Drum Tower Hospital in Nanjing, China. This clinical trial was approved by the Drum Tower Hospital Ethics Committee (2016‐019‐12). The trial is registered with the Australia New Zealand Clinical Trials Registry (ACTRN12614000932662). All participants gave written informed consent for the trial participation and the use of photographs of skin reactions and publication of the results in which individuals could not be identified. Patients acted as their own control and were allocated to use both Mepitel Film and Biafine cream on different sides of their neck. Based on the previous feasibility study, the current sample, using patients as their own controls, was chosen to power the trial to 80% with a *P* < 0.05 and an effect size of 30% (total RISRAS Score); we needed 36 patients to account for a dropout rate of 20%.

### Trial outcomes

Primary trial outcomes were: the effects of Mepitel Film on (1) overall skin reaction severity and (2) the incidence of moist desquamation in head and neck cancer patients treated with radiation therapy.

Secondary trial outcomes were skin dose and patient acceptability of Mepitel Film and Biafine cream.

### Participants

Inclusion criteria included patients receiving radiation therapy for squamous cell carcinoma of the head and neck region in Drum Tower Hospital in Nanjing, China. Specific exclusion criteria included previous radiation therapy to the head and neck region, metastatic disease, facial hair in the research area and a Karnofsky performance status score of less than 70. Patients had to return to the department for four weeks for weekly follow‐up assessments.

### Randomisation

The neck area was divided into a left and right half; each side was randomised to either Mepitel Film or Biafine cream. Both Mepitel Film and Biafine cream were applied from the first day of radiation treatment. Patients were randomised using computer‐generated randomisation charts by PMH.

### Blinding

This trial was not blinded; both researcher and patients knew to which side of the neck the Mepitel Film was applied because of Mepitel Film's visibility and longevity of application.

### Radiation therapy and chemotherapy

Patients received 70–74 Gy in 35–37 fractions to the primary tumour. The areas of interest for this study were the neck node regions, which received 50Gy in 25 fractions. Radiation was delivered using IMRT or tomotherapy with 6MV photons. Concurrent chemotherapy (weekly IV nedaplatin (25mg/m^2^) was given to all patients.

### Application of Mepitel film and Biafine cream

Each patient used both Mepitel Film and Biafine cream on their skin to eliminate confounding patient‐ and treatment‐related factors. Because patients received the same amount of radiation to both sides of their neck, the researcher applied the Mepitel Film to the side of the neck randomised to Mepitel Film and patients applied Biafine cream twice daily to the control side of the neck.^20^ Mepitel Film was replaced if it came off the skin overnight or if significant areas curled up at the edges. Mepitel Film was donated by Mölnlycke Healthcare LTD (Gothenburg, Sweden). The Control Cream, Biafine, was from Johnson & Johnson (France), and contained purified water, liquid paraffin, ethylene glycol monostearate, stearic acid, propylene glycol, paraffin wax, squalane, avocado oil, trolamine/sodium alginate, triethanolamine, cetyl palmitate, methylparaben (sodium salt), sorbic acid (potassium salt), propylparaben (sodium salt) and fragrance. Biafine moisturising cream did not contain sodium lauryl sulphate as this can affect skin barrier function.^21^ Biafine cream is used at Drum Tower Hospital as standard of care. Three studies have shown that Biafine cream is not superior to standard of care.^22^, ^23^, ^24^ Patients were told to apply the Biafine cream twice a day.

### Outcome measure: skin reaction severity

**Figure 1:**
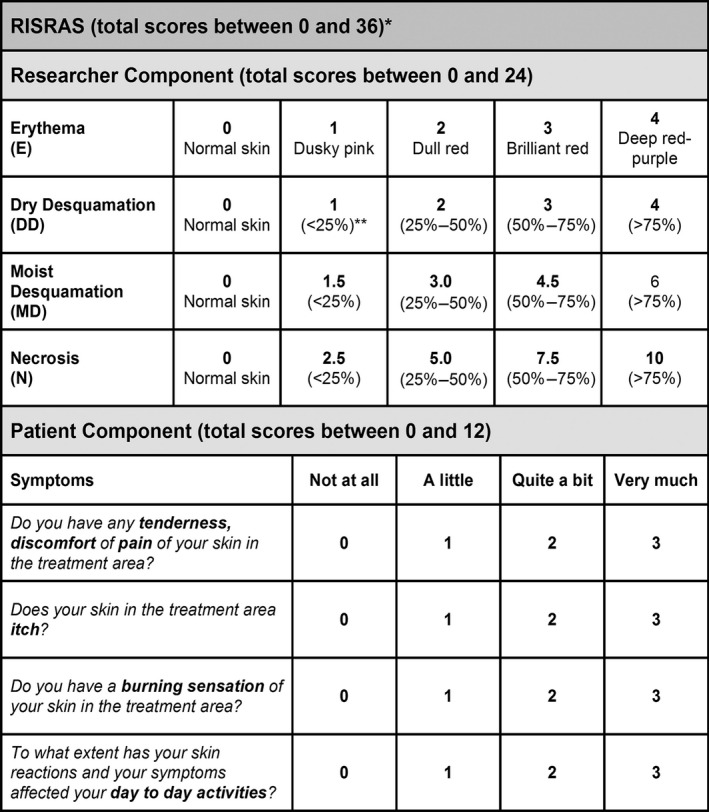
RISRAS scale. The radiation therapist/oncology nurse scores the visible extent of the skin reactions whilst the patient scores the level of pain, itchiness and burning as well as the effect on day‐to‐day life. Summation of these two scores gives the combined RISRAS score.

Skin reaction severity was measured using the modified Radiation‐Induced Skin Reaction Assessment Scale (RISRAS)^25^, ^26^ (Figure 1) and the expanded RTOG scale.^27^, ^28^ RTOG scores were reported by the research radiation therapist as follows; grade 0; no change; grade I: follicular faint or dull erythema/dry desquamation; grade IIA: tender or bright erythema; grade IIB: patchy moist desquamation; and grade III: confluent moist desquamation other than in skinfolds. In order to minimise interscorer variability, we used scorers who were trained in and familiar with RISRAS and expanded RTOG. Scores were determined two or three times a week from day one of radiation therapy to the end of radiation treatment, and after that once a week for four weeks.

### Outcome measure: skin dose

The skin dose of all skin patches covered in Mepitel Film and Biafine cream was measured using gafchromic photographic film, which was applied to both sides of the neck, covering the area where skin reactions were visible.

### Exit questionnaire

All patients were given an exit questionnaire at the end of the trial, to share their thoughts on taking part in the trial and their experience of using Mepitel Film and Biafine cream (see Supplementary Figure 1).

### Statistical analysis

Statistical analysis was conducted using SPSS 15.0 (IBM, Chicago, IL). The statistical significance in differences in dose and skin reaction severity (RISRAS) between Mepitel Film‐ and Biafine cream‐covered skin was determined using the Wilcoxon signed‐rank test. The McNemar test was used to determine the statistical significance of the differences in incidence of moist desquamation between Mepitel Film‐ and Biafine cream‐covered skin. In all cases, *P* < 0.05 was considered statistically significant. Exit questionnaire responses were subjected to a thematic analysis.

## Results

**Figure 2:**
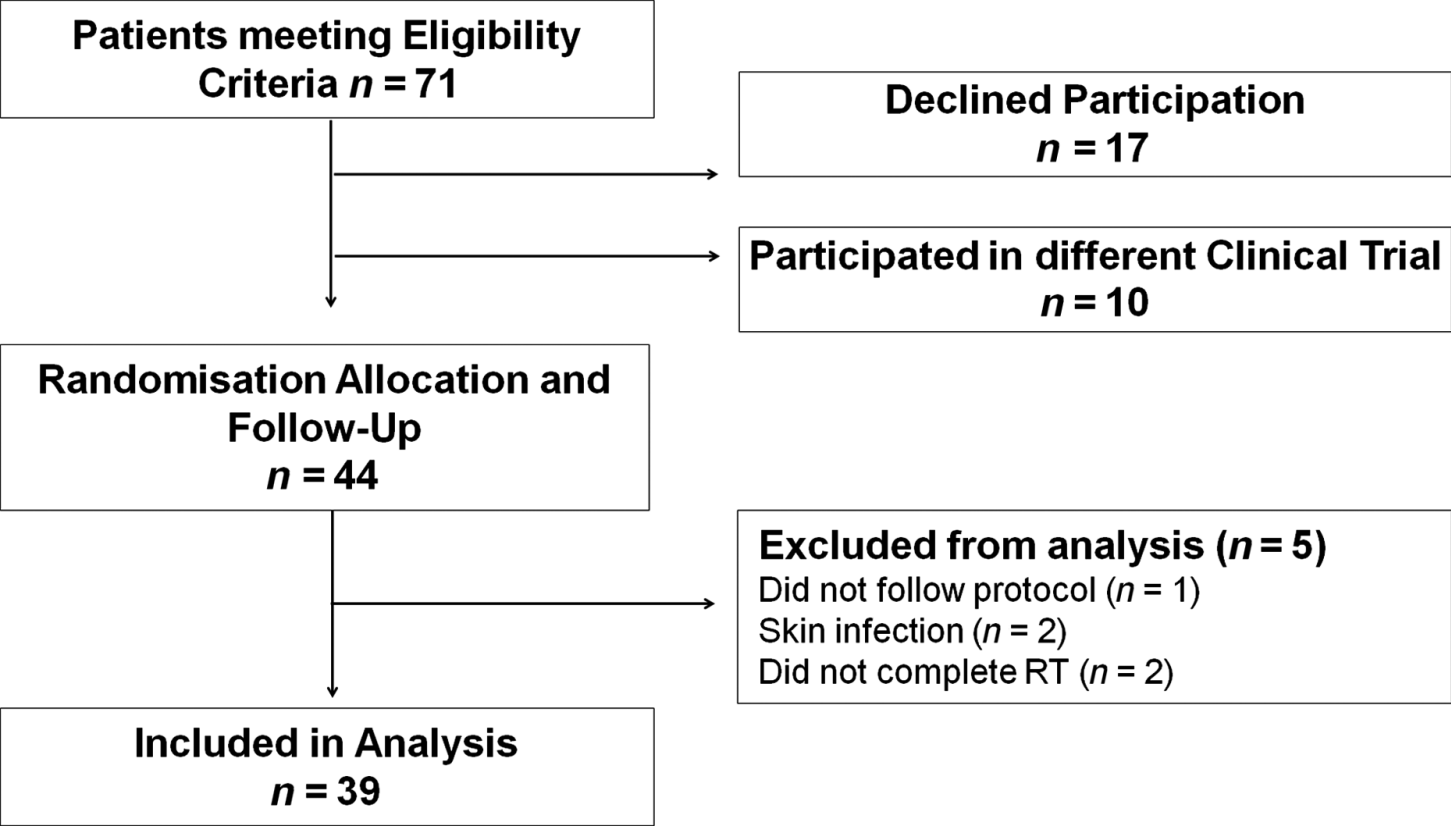
Consort Diagram. Flow of patients through the trial. A total of 71 patients who met eligibility criteria for the trial, 39 patients were included in the analysis. Of these, 11 patient data sets were obtained from the previous feasibility study, with 28 patients contributing new data sets. All patients followed the same prophylactic protocol.

A total of 71 patients were eligible to enter the trial between April 2016 and March 2018 in Drum Tower Hospital in Nanjing, China. However, 10 of these patients were entered in a different head and neck trial. A further 17 patients declined to participate in the trial because they wanted Mepitel Film applied to both sides of their necks. The 44 patients who entered the trial were randomised and had Mepitel Film and Biafine cream allocated to different sides of the neck. Data sets of 5 patients were removed from analysis; one patient was unable to follow protocol, two patients developed a severe skin infection in the control area and two patients did not complete radiation therapy (Figure 2). A total of 39 patients provided full data sets for analysis. Of these, data sets of 11 Chinese patients from the feasibility study were supplemented with data sets from 28 additional Chinese patients, all following the same protocol to meet sample size requirements.

The average age of the cohort was 54 years (range 37–69), with 28 male and 11 female patients. All patients were Epstein–Barr virus (EBV) positive and had grade 3 nasopharyngeal cancer. The vast majority of patients were Asian with Fitzpatrick skin type III.^29^ Tomotherapy was used to deliver radiation to 20 patients and IMRT for 19 patients. Concomitant chemotherapy with weekly IV nedaplatin (25 mg/m^2^) was given to all patients. Table 1 shows the demographic data for this cohort.

**Table 1:** Demographic information of the patient cohort.

Cohort		Number of patients (frequency)
Total enrolled		44
Total completed/analysed		39 (100%)
Sex Male		28 (72%)
Sex: Female		11 (28%)
Average age in years: mean (range)		54 (37–69)
Ethnicity		
European		3 (8%)
Asian		36 (92%)
Cancer type		
SCC Nasopharynx		39 (100%)
Disease grade		
3	39 (100%)	
Disease stage		
I		0 (0%)
II		6 (15%)
III		17 (44%)
IV		16 (41%)
Radiation Therapy		
Total dose to primary tumour		70–74 Gy in 35–37 fractions
Total dose to neck nodes		50 Gy in 25 fractions
IMRT		19 (49%)
Tomotherapy		20 (51%)
Concomitant Chemotherapy		
Nedaplatin		39 (100%)
Fitzpatrick skin type		
II		2 (5%)
III		30 (77%)
IV		7 (18%)
Smoker		
Current		11 (28%)
Previous		1 (3%)
Never		27 (69%)
Alcohol consumption per week		
None		29 (74%)
<1		4 (10%)
1–10		5 (13%)
Sun exposure		
Never/rarely		30 (77%)
Often		9 (23%)

### Comparison of skin dose

Total skin dose is a strong potential confounder of any study that investigates interventions for acute radiation‐induced skin reactions. We used gafchromic film to accurately measure the dose received by all skin patches of all patients. The average dose (± SEM) was 45.1 ± 1.2 Gy for Mepitel Film‐covered skin and 45.2 ± 1.1 Gy for Biafine cream‐covered skin (*P* = 0.925, Wilcoxon signed‐rank test). The strong similarity of skin dose between patches covered in Mepitel Film or Biafine cream means that skin dose was not a confounder of skin severity in this study.

### Comparison of skin reaction severity

**Figure 3:**
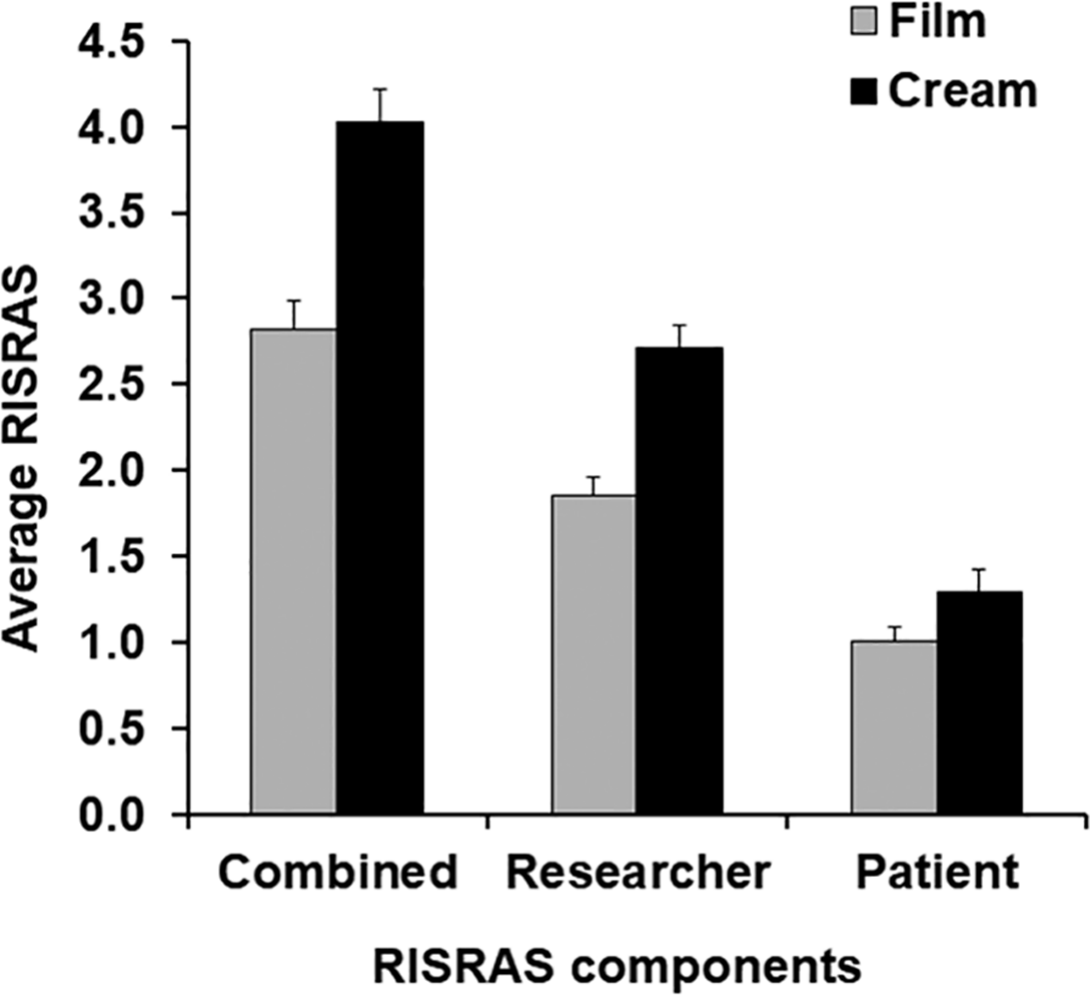
Effects of Mepitel Film and Biafine cream on skin reaction severity, using RISRAS*. *Bar graphs with error bars showing average RISRAS scores plus SEM.

Mepitel Film was applied from the first day of radiation therapy. Biafine cream was applied twice daily. Skin reaction severity under Mepitel Film‐covered and Biafine cream‐covered skin is shown in Figure 3 and Table 2.

**Table 2:** Skin reaction security under Mepitel Film and Biafine cream measured by RISRAS (A) and RTOG (B).

	(A) Average RISRAS scores					
	Mepitel Film (*n* = 39)			Biafine cream (*n* = 39)		
	Combined	Researcher	Patient	Combined	researcher	Patient
Ave ± SEM	2.83 ± 0.16	1.85 ± 0.12	1.00 ± 0.08	4.02 ± 0.20	2.71 ± 0.13	1.29 ± 0.12
% decrease	30	32	23			
*P* value^a^	<0.001	<0.001	0.007			

	(B) RTOG grades				
	I	IIA	IIB	III	MD^b^
Mepitel Film	18 (46%)	13 (33%)	7 (18%)	1 (3%)	8 (21%)
Biafine cream	6 (15%)	9 (23%)	21 (54%)	3 (8%)	24 (62%)

^a^ Wilcoxon signed‐rank test

^b^ MD: Moist desquamation (IIB + III) decrease 41% (*P* < 0.001 McNemar Test)

**Figure 4:**
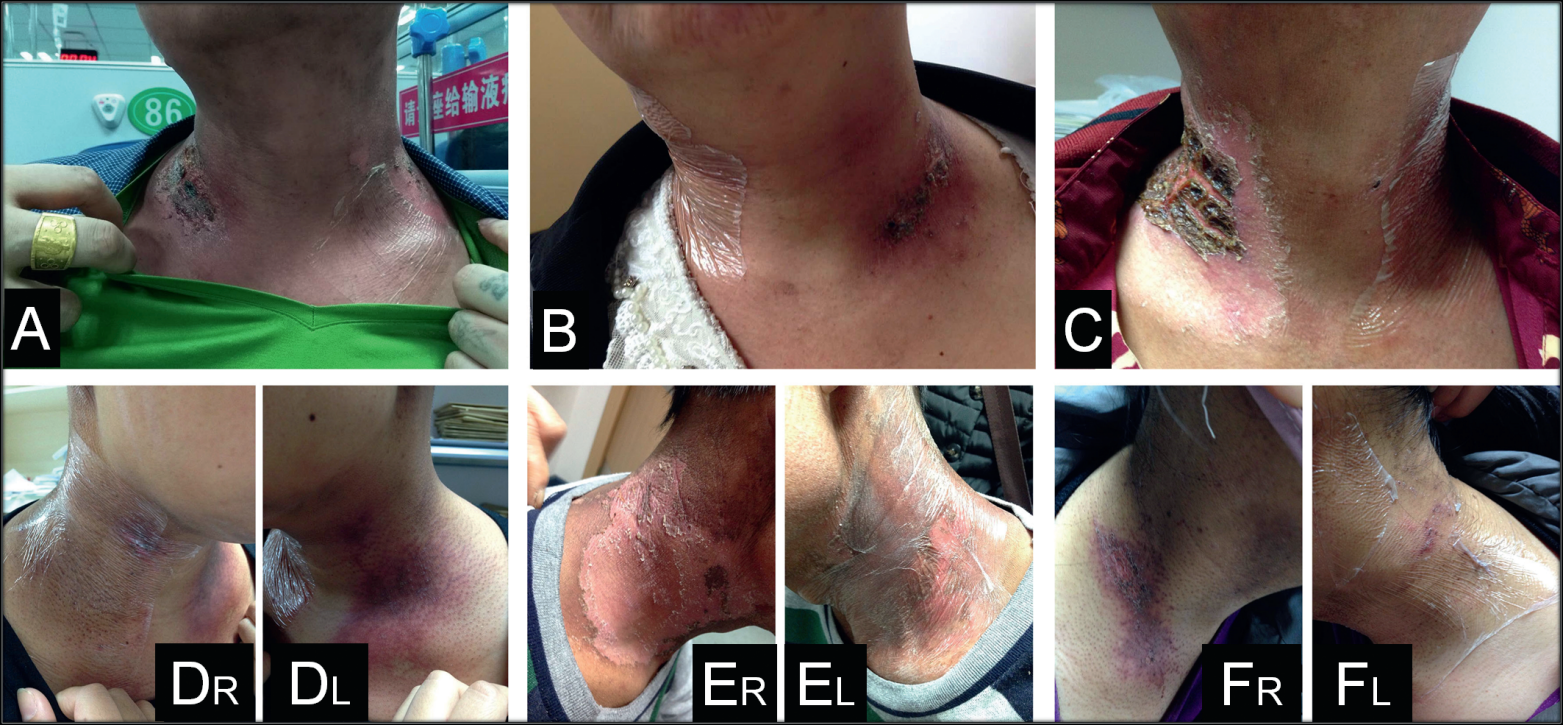
Photographs of skin reaction severity in six patients. Photographs were taken during the final week of treatment (week 5). For patients A–C, a single photograph shows the skin reaction severity on both sides of the neck. For patients D–F, two separate photographs of the right side (R) of the neck and the left side (L) of the neck are included to show skin reaction severity on both sides of the neck. Mepitel Film is visible on one side of the neck of all patients A: DT‐CRT01, 43‐year‐old male; B: DT‐CRT28, 50‐year‐old female; C: DT‐CRT14, 56‐year‐old male; D1 and D2: DT‐CRT13, 37‐year‐old female; E1 and E2: DT‐CRT23, 53‐year‐old male; F1 and F2: DT‐CRT19, 37‐year‐old female.

Bar graphs of average combined, researcher and patients RISRAS scores are shown in Figure 3. Table 2A shows a statistically significant decrease in skin reaction severity for combined, researcher and patients RISRAS components of 30%, 32% and 23%, respectively (*P* < 0.001, 0.001 and 0.007, respectively; Wilcoxon signed‐rank test). Out of 39 patients, four patients scored Mepitel Film worse than Biafine cream in the patient component of RISRAS. Of note, these patients were on the trial at the height of summer with very high temperatures and mentioned that the Mepitel Film often fell off and that the skin underneath the Mepitel Film was itchy. The extended RTOG grading system (Table 2B) revealed a statistically significant 41% decrease in moist desquamation incidence (*P* < 0.001, McNemar test) in favour of Mepitel Film‐covered skin. Photographs were taken of skin reactions on both sides of the neck at least once a week. Figure 4 displays photographs of six of the patients taken during the last two weeks of radiation therapy.

### Acceptability of Mepitel film to patients

All patients filled in the exit questionnaire in which they were asked to comment on their experience with using Mepitel Film (see Supplementary Fig. 1). A content analysis revealed that all the patients found taking part in the trial a positive experience and would take part in future suitable trials. Of the 39 patients, 31 patients (80%) preferred Mepitel Film over Biafine cream with 14 patients (36%) mentioning that Mepitel Film improved symptoms. Eleven patients (28%) mentioned the Mepitel Film did not adhere well enough in the hot weather and in the shower, 16 patients (41%) mentioned the Mepitel Film was uncomfortable to wear on the skin in the summer heat and 12 patients (31%) found the skin under the Mepitel Film quite itchy. An additional three patients (8%) mentioned Mepitel Film felt tight. However, all of these patients remained in the trial.

## Discussion

This intra‐patient controlled unblinded clinical trial assessed whether or not Mepitel Film was superior to Biafine cream in managing the skin reaction severity of head and neck patients undergoing radiation therapy. We randomised 44 Chinese patients and analysed the skin reaction severity in 39 of these patients during and immediately after receiving radiation therapy to the head and neck area. The 39 analysable data sets were made up of 11 from patients in the feasibility study and 28 from additional patients following the exact same protocol, recruited in the same hospital to meet sample size requirements. The results demonstrated that Mepitel Film significantly decreased the severity of acute radiation‐induced skin reactions (as scored by overall RISRAS) by 30% and the incidence of moist desquamation by 41% in 39 head and neck cancer patients. These results confirm the findings from our previous feasibility study.^20^ An earlier trial using Mepilex Lite, a non‐transparent dressing with a foam layer, reported that this dressing decreased time to healing of moist desquamation and improved sleep quality in 88 patients with nasopharyngeal carcinoma.^30^

RISRAS is a very sensitive scale for measuring radiation‐induced skin reactions, as it measures skin reaction severity on a scale of 0–36 (Fig. 1), whereas RTOG grades skin reaction severity on a scale of 0–4. In addition to the visible signs of skin reactions, measured by RTOG and the Researcher Component of RISRAS, we also measured the level of pain, burning, itchiness and effect on day‐to‐day life through the Patient Component of RISRAS and asked patients about their experiences of using Mepitel Film in the Exit Questionnaire.

Two main challenges were encountered in our current trial. Mepitel Film did not adhere well to the skin during hot weather and sometimes in the shower (11/39 patients). The other challenge was that Chinese patients did not tolerate the dressings well during hot weather, with 16/39 Chinese patients finding Mepitel Film uncomfortable and 12/39 patients reporting itchiness underneath Mepitel Film. However, all patients completed the trial.

After we completed our head and neck patient study, two similar clinical trials were published in quick succession. A German trial comparing Mepitel Film with standard care in head and neck cancer patients closed early after 57 patients were randomised because 13 of 28 patients (46.4%) could not tolerate Mepitel Film.^30^ In addition, Mepitel Film adhered poorly in a further 5 patients (18%). A per‐protocol analysis of 9 of 28 Mepitel Film patients and 27 of 27 standard care patients showed that Mepitel Film was not superior to standard care which consisted of fatty cream with 2–5% Urea and Mometasone furoate cream applied 4 times a day. Mometasone furoate is a topical corticosteroid with strong anti‐inflammatory activity combined with relatively low skin thinning activity.^31^ Mometasone furoate has been shown to decrease the severity of radiation‐induced skin reaction severity in several randomised trials.^1^, ^2^, ^4^ These trials suggest that the standard cream in the German study is superior to the standard cream in our studies with respect to reducing skin reaction severity. This and the very low number of patients analysed in the Mepitel Film arm are likely to have contributed to these apparently contradictory results. German patients identified similar problems to the Chinese patients in our head and neck trials: discomfort, itchiness and lack of adherence.

An Australian trial compared a silicone‐film producing gel, StrataXRT, with sorbolene cream for skin reaction severity in 197 head and neck cancer patients in a two‐armed design.^32^ The authors showed that patients treated with StrataXRT had a 12% lower risk of developing grade 2 and a 36% lower risk of developing of grade 3 using the CTCAE v4 scale. StrataXRT was applied by the patients twice a day. StrataXRT seemed well tolerated with a non‐significant decrease in pain and itching and quality of life in patients using Mepitel Film compared with patients using Sorbolene cream.

We have now used soft silicone dressings in five trials, three in breast cancer patients^16^, ^17^, ^18^ and two in head and neck cancer patients^20^ including the current extended trial. We hypothesise that soft silicone dressings, such as Mepitel Film, decrease skin reaction severity by forming a physical barrier against the skin that minimises physical friction damage to the radiation‐damaged skin by items of clothing or other parts of the body. This additional layer of protection gives the skin more opportunity to heal. We have identified two scenarios in which the dressings fail to protect the skin.

Mepitel Film must adhere very closely to the skin to form a physical friction barrier. Too much perspiration and growing stubble pushes the Mepitel Film away from the skin and prevents close adherence. Poor application, whereby the Mepitel Film is not carefully tapped into the ‘nooks and crannies’ of the skin, will result in Mepitel Film sitting on top of the skin, with skin rubbing against skin underneath the Mepitel Film, contributing to further damage.

Mepitel Film will be less effective in preventing skin damage at higher skin doses and will not be effective at all when the skin dose reaches a level that is so high that the skin cannot recover. Mepitel Film decreased skin reaction severity by more than 90% with a complete lack of moist desquamation in 78 breast cancer patients (average skin dose of 30 Gy).^18^ In our head and neck cancer patients, this was by 30% and by 41%, respectively, with average skin dose of 45 Gy. In addition to dose, other factors may have impacted the performance of Mepitel Film in the head and neck cancer cohorts. Skin of the head and neck area is exposed daily to weather and rubs against clothing. This may make it more fragile and more likely to progress to moist desquamation during radiation therapy.^14^

### Strengths and limitations

The biggest strength of this trial is our intra‐patient controlled design: using patients as their own controls minimised possible confounding by treatment and patient‐related factors whilst dose measurements of the skin on both side of the neck of all patients confirmed that dose differences between Mepitel Film and Biafine cream areas were similar and therefore also not a confounding factor in this trial.

The main limitation of this trial was that because of the Mepitel Film's visibility and longevity of application, we were unable to blind the trial. Other limitations included using subjective scales (RISRAS and expanded RTOG) for measuring the skin reaction severity. This means that researcher and patient bias cannot be excluded. In order to minimise, but not exclude completely interscorer variability, the scorers were trained in using both RISRAS and modified RTOG.

Although we asked patients to apply Biafine cream twice a day, we did not try to measure compliance. Biafine cream is very similar to Sorbolene and does not contain sodium lauryl sulphate; three studies have shown that Biafine cream is not superior to standard of care;^22^, ^23^, ^24^ hence, a lack of consistency of Biafine cream usage is unlikely to have confounded the results.

## Conclusions

When used prophylactically, Mepitel Film, compared with Biafine cream, significantly decreased the severity of acute radiation‐induced skin reactions by 30% and moist desquamation rates by 41% in our cohort of 39 Chinese head and neck cancer patients.

## Conflict of interest

The authors have no conflict of interest to declare.

## Trial registration

Australia New Zealand Clinical Trials Registry (ACTRN12614000932662).
